# (Ethane-1,2-di­yl)bis­[bis­(3-methoxy­prop­yl)methyl­phospho­nium] bis­(tetra­phenyl­borate) diethyl ether solvate

**DOI:** 10.1107/S1600536808013962

**Published:** 2008-05-17

**Authors:** Justin L Crossland, Lev N Zakharov, David R Tyler

**Affiliations:** a Department of Chemistry, 1253 University of Oregon, Eugene, Oregon 97403-1253, USA

## Abstract

In the course of substitution studies on the iron dihydrogen complex *trans*-[Fe(DMeOPrPE)_2_(H_2_)H](BPh_4_) {DMeOPrPE = 1,2-bis­[bis­(methoxy­prop­yl)phosphino]ethane}, we discovered an unexpected transformation of the diphosphine ligand to a diphospho­nium dication without the use of any typical methyl­ating reagent. The P atoms in the dication of the title compound, C_20_H_46_O_4_P_2_^2+^·2C_24_H_20_B^−^·C_4_H_10_O, have a distorted tetra­hedral coordination with P—C(Me) distances of 1.791 (2) and 1.785 (2) Å. The P—C—C—P torsion angle about the central dimethyl­ene bridge is −168.3 (1)°.

## Related literature

For related literature, see: Churchill *et al.* (1990 ▶); Crossland *et al.* (2007 ▶); Gilbertson *et al.* (2005 ▶, 2007 ▶); Miller *et al.* (2002 ▶); Szymczak *et al.* (2007 ▶); van der Sluis & Spek (1990 ▶).
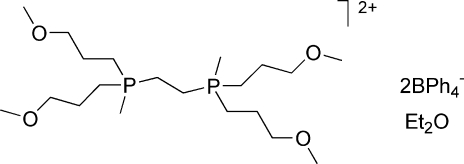


## Experimental

### 

#### Crystal data

C_20_H_46_O_4_P_2_^2+^·2C_24_H_20_B^−^·C_4_H_10_O
*M* *_r_* = 1125.1
Orthorhombic, 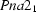

*a* = 39.548 (4) Å
*b* = 12.6038 (11) Å
*c* = 13.1767 (12) Å
*V* = 6567.9 (10) Å^3^
*Z* = 4
Mo *K*α radiation
μ = 0.13 mm^−1^
*T* = 173 (2) K
0.38 × 0.36 × 0.14 mm

#### Data collection

Bruker SMART APEX CCD area-detector diffractometer
Absorption correction: multi-scan (*SADABS*; Sheldrick, 1995 ▶; Blessing, 1995 ▶) *T*_min_ = 0.952, *T*_max_ = 0.982
71930 measured reflections
14334 independent reflections
12932 reflections with *I* > 2σ(*I*)
*R*_int_ = 0.024

#### Refinement

*R*[*F* ^2^ > 2σ(*F* ^2^)] = 0.043
*wR*(*F* ^2^) = 0.114
*S* = 1.01
14334 reflections
685 parameters
1 restraint
H-atom parameters constrained
Δρ_max_ = 0.37 e Å^−3^
Δρ_min_ = −0.14 e Å^−3^
Absolute structure: Flack (1983 ▶); 6847 Friedel pairs
Flack parameter: 0.00 (5)

### 

Data collection: *SMART* (Bruker, 2000 ▶); cell refinement: *SAINT* (Bruker, 2000 ▶); data reduction: *SAINT*; program(s) used to solve structure: *SHELXTL* (Sheldrick, 2008 ▶); program(s) used to refine structure: *SHELXTL*; molecular graphics: *SHELXTL*; software used to prepare material for publication: *SHELXTL*.

## Supplementary Material

Crystal structure: contains datablocks I, global. DOI: 10.1107/S1600536808013962/ya2073sup1.cif

Structure factors: contains datablocks I. DOI: 10.1107/S1600536808013962/ya2073Isup2.hkl

Additional supplementary materials:  crystallographic information; 3D view; checkCIF report

## Figures and Tables

**Fig. 1.**
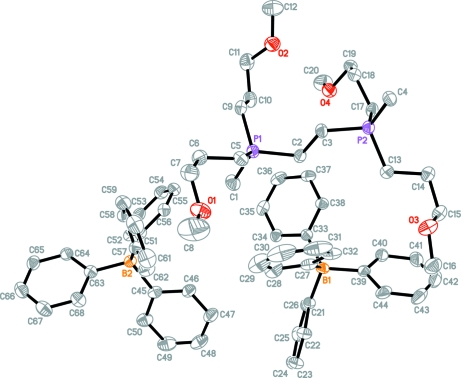
The structure of the title compound with 30% probability displacement ellipsoids and the atom-numbering scheme. The H atoms are omitted for clarity; disordered Et2O molecule was not located, and therefore cannot be shown.

## supplementary crystallographic information

### Comment

The bidentate diphosphine ligand 1,2-bis[di(methoxypropyl)phosphino]ethane (DMeOPrPE) has been used in the synthesis of numerous transition metal complexes (Miller *et al.*, 2002; Gilbertson *et al.*, 2005; Crossland *et al.*, 2007). The strong donating ability of this ligand allows these complexes to bind weakly coordinating molecules such as H_2_ and N_2_ (Gilbertson *et al.*, 2007; Szymczak *et al.*, 2007)). The DMeOPrPE ligand also imparts diverse solubility; often the complexes are soluble in solvents ranging from hexane to water. In the course of substitution studies on iron dihydrogen complexes, specifically *trans*-[Fe(DMeOPrPE)_2_(H_2_)H]^+^, we discovered an unexpected transformation of the diphosphine ligand to a phosphonium dication without the use of any typical methylating reagent.  At this point the mechanism, by which this diphosphonium is formed, is unclear. We postulate that oxygen atom coordination of a methoxypropyl arm of the DMeOPrPE ligand to the iron center could activate the terminal methyl group for nucleophilic attack by another DMeOPrPE ligand; however, we currently have no evidence for such a mechanism.  Alternatively, coordination of acetonitrile to the metal center could activate the methyl group toward nucleophilic attack by the phosphine ligand.

The P atoms in the dication of the title compound [(DMeOPrPE)Me_2_]^2+^[BPh_4_]_2_^-^.Et_2_O have a distorted tetrahedral coordination with P-C(Me) distances of 1.791 (2) and 1.785 (2) Å. The torsion angle P1-C2-C3-P2 over the central dimethylene bridge is equal to -168.3 (1)°.

### Experimental

The dihydrogen complex, *trans*-[Fe(DMeOPrPE)_2_H(H_2_)](BPh_4_) (DMeOPrPE = 1,2-bis[di(methoxypropyl)phosphino]ethane), was prepared by reported procedures, using NaBPh_4_ instead of TlPF_6_ (Gilbertson *et al.*, 2007). This complex (0.057 g, 0.053 mmol) was then reacted with the excess of MeCN (0.28 mL, 5.3 mmol) to form *trans*-[Fe(DMeOPrPE)_2_(CH_3_CN)H][BPh_4_] after 12 hrs (Gilbertson *et al.*,2007).  The acetonitrile complex was not isolated. The resulting solution was layered with Et_2_O and allowed to stand at room temperature for 2 months. The title compound formed as pale yellow plates and gave a single ^31^P{^1^H} NMR resonance at 38.7 ppm. A ^31^P{^1^H} NMR spectrum of the mother liquor revealed two major resonances at 80.9 ppm. and 63.7 ppm., assignable to *trans*-[Fe(DMeOPrPE)_2_(CH_3_CN)H]^+^ and *trans*-[Fe(DMeOPrPE)_2_(CH_3_CN)_2_]^2+^ respectively, as well as a minor amount of the title compound.

### Refinement

A highly disordered solvent molecule, most probably Et_2_O, was found to be present in crystal; however our attempts to locate the individual atoms were unsuccessful. Therefore, in order to take into account the contribution of the disordered solvent, we applied the SQUEEZE technique (Van der Sluis & Spek 1990). Correction of the X-ray data by SQUEEZE (155 electrons/cell) was close to the expected value for four Et_2_O molecules per unit cell (168 electrons/cell). The H atoms were positioned geometrically and refined in the riding model approximation, C—H = 0.95, 0.99 and 0.98 Å; *U*_iso_(H) = 1.2*U*_eq_(C) and 1.5*U*_eq_(C), respectively for –CH, –CH_2_ and –CH_3_ groups.

### Crystal data

**Table:**

C_20_H_46_O_4_P_2_^2+^·2(C_24_H_20_B_1_^–^)·C_4_H_10_O_1_	*F*_000_ = 2432
*M**_r_* = 1125.1	*D*_x_ = 1.275 Mg m^−^^3^
Orthorhombic, *P**n**a*2_1_	Mo *K*α radiation λ = 0.71073 Å
Hall symbol: P 2c -2n	Cell parameters from 7356 reflections
*a* = 39.548 (4) Å	θ = 2.2–26.3º
*b* = 12.6038 (11) Å	µ = 0.13 mm^−^^1^
*c* = 13.1767 (12) Å	*T* = 173 (2) K
*V* = 6567.9 (10) Å^3^	Plate, light yellow
*Z* = 4	0.38 × 0.36 × 0.14 mm

### Data collection

**Table:**

Bruker SMART APEX CCD area-detector diffractometer	14334 independent reflections
Radiation source: fine-focus sealed tube	12932 reflections with *I* > 2σ(*I*)
Monochromator: graphite	*R*_int_ = 0.024
*T* = 173(2) K	θ_max_ = 27.0º
φ and ω scans	θ_min_ = 1.0º
Absorption correction: multi-scan(SADABS; Sheldrick, 1995; Blessing, 1995)	*h* = −50→50
*T*_min_ = 0.952, *T*_max_ = 0.982	*k* = −16→16
71930 measured reflections	*l* = −16→16

### Refinement

**Table:**

Refinement on *F*^2^	Hydrogen site location: inferred from neighbouring sites
Least-squares matrix: full	H-atom parameters constrained
*R*[*F*^2^ > 2σ(*F*^2^)] = 0.043	*w* = 1/[σ^2^(*F*_o_^2^) + (0.0832*P*)^2^] where *P* = (*F*_o_^2^ + 2*F*_c_^2^)/3
*wR*(*F*^2^) = 0.114	(Δ/σ)_max_ = 0.003
*S* = 1.01	Δρ_max_ = 0.37 e Å^−^^3^
14334 reflections	Δρ_min_ = −0.14 e Å^−^^3^
685 parameters	Extinction correction: none
1 restraint	Absolute structure: Flack (1983); 6847 Friedel pairs
Primary atom site location: structure-invariant direct methods	Flack parameter: 0.00 (5)
Secondary atom site location: difference Fourier map	

### Special details

**Table:**

Geometry. All e.s.d.'s (except the e.s.d. in the dihedral angle between two l.s. planes)are estimated using the full covariance matrix. The cell e.s.d.'s are takeninto account individually in the estimation of e.s.d.'s in distances, anglesand torsion angles; correlations between e.s.d.'s in cell parameters are onlyused when they are defined by crystal symmetry. An approximate (isotropic)treatment of cell e.s.d.'s is used for estimating e.s.d.'s involving l.s. planes.
Refinement. Refinement of *F*^2^ against ALL reflections. The weighted *R*-factor *wR* and goodness of fit *S* are based on *F*^2^, conventional *R*-factors *R* are based on *F*, with *F* set to zero for negative *F*^2^. The threshold expression of *F*^2^ > σ(*F*^2^) is used only for calculating *R*-factors(gt) *etc*. and is not relevant to the choice of reflections for refinement. *R*-factors based on *F*^2^ are statistically about twice as large as those based on *F*, and *R*-factors based on ALL data will be even larger.

### Fractional atomic coordinates and isotropic or equivalent isotropic displacement parameters (Å^2^)

**Table:**

	*x*	*y*	*z*	*U*_iso_*/*U*_eq_	
P1	0.911570 (10)	0.94306 (4)	0.24530 (3)	0.04314 (10)	
P2	0.832165 (10)	0.95915 (3)	0.00836 (3)	0.04089 (10)	
O1	0.97349 (4)	0.65112 (11)	0.23890 (14)	0.0727 (4)	
O2	0.91181 (4)	1.26782 (11)	0.11308 (12)	0.0619 (3)	
O3	0.77829 (4)	0.64990 (11)	−0.00135 (12)	0.0653 (4)	
O4	0.82537 (4)	1.25529 (10)	0.18899 (10)	0.0557 (3)	
B1	0.79371 (5)	0.74618 (15)	0.42316 (13)	0.0412 (4)	
B2	0.94798 (4)	0.83084 (15)	0.77964 (14)	0.0390 (4)	
C1	0.90670 (5)	0.92107 (19)	0.37877 (15)	0.0600 (5)	
H1A	0.9288	0.9261	0.4120	0.090*	
H1B	0.8971	0.8503	0.3902	0.090*	
H1C	0.8915	0.9748	0.4071	0.090*	
C2	0.87039 (4)	0.93324 (15)	0.18730 (13)	0.0453 (4)	
H2A	0.8600	0.8645	0.2060	0.054*	
H2B	0.8557	0.9905	0.2138	0.054*	
C3	0.87232 (4)	0.94183 (14)	0.07080 (13)	0.0435 (4)	
H3A	0.8831	0.8768	0.0440	0.052*	
H3B	0.8871	1.0025	0.0531	0.052*	
C4	0.84123 (5)	0.95670 (15)	−0.12429 (14)	0.0489 (4)	
H4A	0.8202	0.9659	−0.1627	0.073*	
H4B	0.8516	0.8885	−0.1421	0.073*	
H4C	0.8569	1.0144	−0.1409	0.073*	
C5	0.93830 (4)	0.84373 (15)	0.19051 (14)	0.0489 (4)	
H5A	0.9272	0.7738	0.1986	0.059*	
H5B	0.9403	0.8578	0.1168	0.059*	
C6	0.97363 (5)	0.83772 (15)	0.2358 (2)	0.0586 (5)	
H6A	0.9868	0.9006	0.2146	0.070*	
H6B	0.9721	0.8383	0.3108	0.070*	
C7	0.99146 (5)	0.73853 (18)	0.2017 (2)	0.0682 (6)	
H7A	0.9924	0.7360	0.1266	0.082*	
H7B	1.0149	0.7376	0.2280	0.082*	
C8	0.98754 (12)	0.5538 (3)	0.2070 (4)	0.1449 (17)	
H8A	0.9744	0.4951	0.2356	0.217*	
H8B	1.0110	0.5489	0.2305	0.217*	
H8C	0.9870	0.5498	0.1328	0.217*	
C9	0.92862 (4)	1.07202 (14)	0.22043 (15)	0.0482 (4)	
H9A	0.9516	1.0759	0.2502	0.058*	
H9B	0.9310	1.0808	0.1461	0.058*	
C10	0.90770 (5)	1.16456 (17)	0.26171 (15)	0.0558 (5)	
H10A	0.8836	1.1532	0.2451	0.067*	
H10B	0.9099	1.1672	0.3365	0.067*	
C11	0.91921 (6)	1.26808 (16)	0.21734 (18)	0.0644 (5)	
H11A	0.9074	1.3277	0.2510	0.077*	
H11B	0.9438	1.2770	0.2279	0.077*	
C12	0.92053 (8)	1.36238 (19)	0.0625 (2)	0.0864 (8)	
H12A	0.9146	1.3564	−0.0094	0.130*	
H12B	0.9449	1.3746	0.0690	0.130*	
H12C	0.9082	1.4219	0.0929	0.130*	
C13	0.80570 (4)	0.84972 (14)	0.04360 (14)	0.0475 (4)	
H13A	0.8192	0.7838	0.0389	0.057*	
H13B	0.7991	0.8587	0.1156	0.057*	
C14	0.77376 (5)	0.83502 (16)	−0.01878 (17)	0.0559 (5)	
H14A	0.7586	0.8968	−0.0093	0.067*	
H14B	0.7796	0.8300	−0.0917	0.067*	
C15	0.75584 (5)	0.73456 (16)	0.01469 (17)	0.0575 (5)	
H15A	0.7497	0.7392	0.0874	0.069*	
H15B	0.7349	0.7241	−0.0254	0.069*	
C16	0.76678 (6)	0.55335 (17)	0.03814 (19)	0.0679 (6)	
H16A	0.7834	0.4976	0.0238	0.102*	
H16B	0.7451	0.5348	0.0066	0.102*	
H16C	0.7637	0.5600	0.1117	0.102*	
C17	0.81313 (4)	1.08180 (14)	0.04768 (14)	0.0459 (4)	
H17A	0.7915	1.0914	0.0109	0.055*	
H17B	0.8080	1.0779	0.1211	0.055*	
C18	0.83580 (4)	1.17816 (13)	0.02792 (14)	0.0480 (4)	
H18A	0.8591	1.1617	0.0500	0.058*	
H18B	0.8363	1.1931	−0.0458	0.058*	
C19	0.82331 (5)	1.27586 (14)	0.08396 (15)	0.0510 (4)	
H19A	0.8374	1.3380	0.0663	0.061*	
H19B	0.7996	1.2915	0.0646	0.061*	
C20	0.81650 (8)	1.34563 (19)	0.2477 (2)	0.0839 (7)	
H20A	0.8181	1.3282	0.3200	0.126*	
H20B	0.7933	1.3669	0.2316	0.126*	
H20C	0.8320	1.4041	0.2320	0.126*	
C21	0.78618 (4)	0.68610 (13)	0.53154 (12)	0.0420 (3)	
C22	0.79233 (5)	0.57695 (15)	0.54487 (16)	0.0559 (4)	
H22A	0.8030	0.5384	0.4918	0.067*	
C23	0.78333 (6)	0.52385 (18)	0.63310 (18)	0.0669 (6)	
H23A	0.7876	0.4499	0.6389	0.080*	
C24	0.76838 (5)	0.5769 (2)	0.71195 (16)	0.0659 (6)	
H24A	0.7629	0.5408	0.7730	0.079*	
C25	0.76143 (5)	0.6835 (2)	0.70145 (14)	0.0586 (5)	
H25A	0.7507	0.7208	0.7552	0.070*	
C26	0.76999 (4)	0.73713 (16)	0.61236 (13)	0.0467 (4)	
H26A	0.7647	0.8104	0.6064	0.056*	
C27	0.82566 (5)	0.69210 (13)	0.36231 (15)	0.0508 (4)	
C28	0.85635 (6)	0.67357 (19)	0.4118 (2)	0.0719 (6)	
H28A	0.8581	0.6869	0.4826	0.086*	
C29	0.88499 (7)	0.6353 (2)	0.3584 (3)	0.0974 (10)	
H29A	0.9058	0.6257	0.3934	0.117*	
C30	0.88308 (10)	0.6121 (2)	0.2578 (3)	0.1037 (12)	
H30A	0.9021	0.5847	0.2225	0.124*	
C31	0.85326 (10)	0.6292 (2)	0.2087 (2)	0.0975 (11)	
H31A	0.8517	0.6131	0.1384	0.117*	
C32	0.82511 (7)	0.66919 (15)	0.25845 (16)	0.0648 (6)	
H32A	0.8050	0.6814	0.2210	0.078*	
C33	0.80584 (4)	0.86990 (13)	0.43980 (12)	0.0380 (3)	
C34	0.82293 (4)	0.90416 (14)	0.52690 (13)	0.0441 (3)	
H34A	0.8264	0.8552	0.5807	0.053*	
C35	0.83506 (4)	1.00754 (16)	0.53765 (16)	0.0531 (4)	
H35A	0.8464	1.0276	0.5983	0.064*	
C36	0.83078 (5)	1.08061 (15)	0.46135 (18)	0.0586 (5)	
H36A	0.8389	1.1511	0.4689	0.070*	
C37	0.81437 (5)	1.04982 (14)	0.37331 (16)	0.0505 (4)	
H37A	0.8112	1.0993	0.3198	0.061*	
C38	0.80252 (4)	0.94648 (13)	0.36320 (13)	0.0418 (3)	
H38A	0.7917	0.9269	0.3016	0.050*	
C39	0.75804 (5)	0.73372 (14)	0.36048 (12)	0.0461 (4)	
C40	0.73388 (5)	0.81338 (17)	0.35444 (14)	0.0548 (5)	
H40A	0.7385	0.8801	0.3850	0.066*	
C41	0.70291 (5)	0.7988 (2)	0.30485 (17)	0.0711 (6)	
H41A	0.6868	0.8547	0.3032	0.085*	
C42	0.69587 (7)	0.7036 (3)	0.25857 (16)	0.0845 (9)	
H42A	0.6752	0.6942	0.2230	0.101*	
C43	0.71877 (8)	0.6227 (3)	0.26394 (17)	0.0812 (8)	
H43A	0.7139	0.5565	0.2327	0.097*	
C44	0.74898 (6)	0.63734 (18)	0.31486 (14)	0.0642 (6)	
H44A	0.7643	0.5794	0.3191	0.077*	
C45	0.91392 (4)	0.76182 (13)	0.80706 (14)	0.0446 (4)	
C46	0.88717 (4)	0.75319 (14)	0.73721 (16)	0.0506 (4)	
H46A	0.8898	0.7842	0.6720	0.061*	
C47	0.85679 (5)	0.70099 (17)	0.75936 (19)	0.0630 (5)	
H47A	0.8394	0.6967	0.7098	0.076*	
C48	0.85237 (6)	0.6561 (2)	0.8534 (2)	0.0768 (7)	
H48A	0.8319	0.6203	0.8695	0.092*	
C49	0.87768 (7)	0.6633 (2)	0.92349 (19)	0.0771 (7)	
H49A	0.8745	0.6326	0.9886	0.093*	
C50	0.90827 (6)	0.71519 (18)	0.90178 (16)	0.0599 (5)	
H50A	0.9254	0.7186	0.9523	0.072*	
C51	0.93524 (4)	0.95472 (13)	0.78414 (13)	0.0401 (3)	
C52	0.93251 (4)	1.00970 (13)	0.87662 (13)	0.0421 (3)	
H52A	0.9399	0.9753	0.9369	0.051*	
C53	0.91954 (4)	1.11195 (14)	0.88415 (16)	0.0521 (4)	
H53A	0.9187	1.1467	0.9481	0.062*	
C54	0.90790 (5)	1.16254 (15)	0.79823 (18)	0.0590 (5)	
H54A	0.8987	1.2320	0.8026	0.071*	
C55	0.90968 (5)	1.11160 (17)	0.70600 (17)	0.0581 (5)	
H55A	0.9018	1.1461	0.6464	0.070*	
C56	0.92309 (4)	1.00913 (14)	0.69979 (14)	0.0489 (4)	
H56A	0.9239	0.9753	0.6354	0.059*	
C57	0.96259 (4)	0.79928 (13)	0.66663 (12)	0.0413 (3)	
C58	0.98485 (4)	0.86591 (16)	0.61458 (14)	0.0498 (4)	
H58A	0.9890	0.9348	0.6410	0.060*	
C59	1.00115 (5)	0.83621 (18)	0.52648 (15)	0.0604 (5)	
H59A	1.0163	0.8842	0.4942	0.072*	
C60	0.99563 (6)	0.7370 (2)	0.48486 (16)	0.0672 (6)	
H60A	1.0068	0.7163	0.4242	0.081*	
C61	0.97359 (6)	0.66888 (19)	0.53315 (17)	0.0697 (6)	
H61A	0.9694	0.6006	0.5054	0.084*	
C62	0.95753 (5)	0.69979 (15)	0.62239 (15)	0.0556 (4)	
H62A	0.9425	0.6514	0.6544	0.067*	
C63	0.97989 (4)	0.80468 (14)	0.85571 (12)	0.0425 (3)	
C64	1.00050 (4)	0.88009 (15)	0.90241 (14)	0.0465 (4)	
H64A	0.9953	0.9530	0.8929	0.056*	
C65	1.02823 (5)	0.85380 (17)	0.96220 (15)	0.0559 (5)	
H65A	1.0409	0.9082	0.9944	0.067*	
C66	1.03732 (6)	0.7494 (2)	0.97483 (16)	0.0654 (5)	
H66A	1.0564	0.7309	1.0149	0.078*	
C67	1.01830 (6)	0.67244 (18)	0.92838 (17)	0.0645 (5)	
H67A	1.0244	0.5999	0.9354	0.077*	
C68	0.99007 (5)	0.69976 (16)	0.87099 (15)	0.0560 (4)	
H68A	0.9771	0.6446	0.8409	0.067*	

### Atomic displacement parameters (Å^2^)

**Table:**

	*U*^11^	*U*^22^	*U*^33^	*U*^12^	*U*^13^	*U*^23^
P1	0.0388 (2)	0.0519 (2)	0.0387 (2)	−0.00045 (17)	−0.00240 (17)	0.00356 (19)
P2	0.03627 (19)	0.0431 (2)	0.0433 (2)	0.00072 (16)	−0.00429 (16)	0.00035 (18)
O1	0.0891 (11)	0.0520 (8)	0.0770 (10)	0.0136 (7)	0.0054 (9)	0.0073 (8)
O2	0.0683 (8)	0.0546 (8)	0.0627 (8)	−0.0045 (6)	−0.0029 (7)	0.0002 (7)
O3	0.0639 (8)	0.0541 (7)	0.0778 (10)	−0.0116 (6)	0.0177 (8)	−0.0065 (7)
O4	0.0677 (8)	0.0461 (7)	0.0532 (7)	0.0001 (6)	−0.0047 (6)	−0.0017 (5)
B1	0.0502 (10)	0.0412 (9)	0.0322 (8)	−0.0024 (7)	0.0039 (7)	0.0053 (7)
B2	0.0399 (9)	0.0411 (9)	0.0361 (8)	−0.0016 (7)	0.0052 (7)	−0.0023 (7)
C1	0.0598 (11)	0.0806 (13)	0.0396 (10)	0.0023 (10)	0.0010 (8)	0.0039 (9)
C2	0.0385 (8)	0.0544 (10)	0.0429 (9)	−0.0076 (7)	−0.0023 (7)	0.0015 (7)
C3	0.0364 (8)	0.0508 (9)	0.0433 (9)	0.0032 (6)	−0.0034 (7)	0.0011 (7)
C4	0.0476 (9)	0.0553 (10)	0.0438 (9)	0.0032 (7)	−0.0036 (7)	0.0036 (8)
C5	0.0518 (9)	0.0498 (9)	0.0451 (9)	0.0073 (7)	−0.0006 (8)	0.0059 (7)
C6	0.0468 (9)	0.0514 (10)	0.0777 (13)	0.0022 (8)	−0.0011 (9)	0.0101 (10)
C7	0.0540 (11)	0.0691 (13)	0.0814 (15)	0.0128 (10)	0.0136 (10)	0.0136 (11)
C8	0.173 (4)	0.0621 (18)	0.200 (5)	0.030 (2)	0.039 (4)	−0.005 (2)
C9	0.0389 (8)	0.0536 (10)	0.0522 (10)	−0.0035 (7)	−0.0080 (7)	0.0002 (7)
C10	0.0556 (10)	0.0620 (11)	0.0497 (11)	0.0056 (9)	−0.0068 (8)	−0.0098 (9)
C11	0.0674 (12)	0.0514 (11)	0.0743 (15)	0.0021 (9)	−0.0145 (10)	−0.0151 (10)
C12	0.107 (2)	0.0571 (13)	0.0949 (19)	−0.0025 (13)	0.0167 (16)	0.0055 (13)
C13	0.0488 (9)	0.0464 (9)	0.0471 (9)	−0.0060 (7)	−0.0026 (7)	0.0019 (7)
C14	0.0454 (9)	0.0579 (11)	0.0645 (12)	−0.0075 (8)	−0.0052 (8)	0.0037 (9)
C15	0.0451 (9)	0.0611 (11)	0.0663 (12)	−0.0101 (8)	0.0012 (9)	−0.0034 (10)
C16	0.0775 (14)	0.0562 (12)	0.0699 (13)	−0.0145 (10)	−0.0011 (11)	−0.0071 (10)
C17	0.0361 (8)	0.0459 (9)	0.0555 (10)	0.0039 (7)	−0.0053 (7)	−0.0041 (8)
C18	0.0481 (9)	0.0475 (9)	0.0485 (10)	−0.0025 (7)	−0.0029 (7)	0.0046 (7)
C19	0.0499 (9)	0.0429 (9)	0.0603 (11)	0.0014 (7)	−0.0049 (8)	0.0102 (8)
C20	0.120 (2)	0.0553 (12)	0.0764 (15)	−0.0084 (13)	0.0048 (15)	−0.0167 (12)
C21	0.0392 (8)	0.0483 (9)	0.0383 (8)	−0.0048 (6)	0.0001 (6)	0.0100 (7)
C22	0.0640 (11)	0.0496 (10)	0.0541 (11)	−0.0055 (8)	−0.0008 (9)	0.0147 (8)
C23	0.0703 (13)	0.0634 (12)	0.0668 (13)	−0.0122 (10)	−0.0102 (11)	0.0319 (11)
C24	0.0572 (11)	0.0902 (16)	0.0502 (11)	−0.0231 (11)	−0.0071 (9)	0.0335 (11)
C25	0.0423 (9)	0.0958 (16)	0.0378 (9)	−0.0116 (9)	0.0033 (7)	0.0078 (9)
C26	0.0388 (8)	0.0641 (11)	0.0370 (8)	−0.0046 (7)	0.0009 (6)	0.0071 (7)
C27	0.0618 (11)	0.0352 (8)	0.0552 (10)	−0.0068 (7)	0.0196 (9)	0.0024 (7)
C28	0.0599 (12)	0.0653 (13)	0.0906 (17)	0.0035 (10)	0.0155 (12)	−0.0102 (12)
C29	0.0667 (15)	0.0654 (15)	0.160 (3)	0.0063 (12)	0.0275 (18)	−0.0080 (18)
C30	0.112 (2)	0.0573 (14)	0.142 (3)	−0.0062 (15)	0.082 (2)	−0.0172 (16)
C31	0.149 (3)	0.0531 (13)	0.091 (2)	−0.0150 (16)	0.078 (2)	−0.0080 (12)
C32	0.0977 (15)	0.0426 (9)	0.0542 (11)	−0.0125 (10)	0.0318 (11)	0.0013 (8)
C33	0.0351 (7)	0.0420 (8)	0.0370 (7)	0.0012 (6)	0.0048 (6)	0.0030 (6)
C34	0.0391 (8)	0.0499 (9)	0.0432 (9)	−0.0023 (7)	0.0009 (7)	−0.0008 (7)
C35	0.0428 (9)	0.0585 (11)	0.0580 (11)	−0.0053 (8)	0.0003 (8)	−0.0125 (9)
C36	0.0510 (10)	0.0417 (9)	0.0829 (14)	−0.0062 (8)	0.0155 (10)	−0.0088 (9)
C37	0.0476 (9)	0.0416 (9)	0.0623 (11)	0.0023 (7)	0.0140 (8)	0.0105 (8)
C38	0.0401 (8)	0.0447 (9)	0.0406 (8)	0.0002 (6)	0.0075 (7)	0.0037 (7)
C39	0.0552 (10)	0.0544 (10)	0.0287 (7)	−0.0181 (8)	0.0010 (7)	0.0085 (7)
C40	0.0512 (10)	0.0719 (13)	0.0414 (9)	−0.0164 (9)	−0.0022 (7)	0.0140 (9)
C41	0.0541 (11)	0.1074 (18)	0.0519 (11)	−0.0160 (11)	−0.0026 (9)	0.0276 (12)
C42	0.0745 (15)	0.134 (2)	0.0448 (11)	−0.0572 (17)	−0.0104 (10)	0.0147 (13)
C43	0.0920 (18)	0.1022 (19)	0.0494 (12)	−0.0607 (17)	0.0043 (11)	−0.0037 (12)
C44	0.0842 (14)	0.0668 (12)	0.0417 (9)	−0.0351 (11)	0.0050 (10)	0.0037 (9)
C45	0.0461 (9)	0.0415 (8)	0.0462 (9)	−0.0031 (7)	0.0137 (7)	−0.0049 (7)
C46	0.0441 (8)	0.0482 (9)	0.0595 (10)	−0.0045 (7)	0.0077 (8)	−0.0005 (8)
C47	0.0452 (10)	0.0592 (11)	0.0846 (15)	−0.0089 (8)	0.0055 (10)	−0.0009 (10)
C48	0.0582 (12)	0.0794 (15)	0.0928 (18)	−0.0217 (11)	0.0256 (12)	0.0010 (13)
C49	0.0868 (16)	0.0843 (16)	0.0603 (13)	−0.0124 (13)	0.0325 (12)	0.0110 (11)
C50	0.0713 (12)	0.0618 (11)	0.0465 (10)	−0.0086 (9)	0.0148 (9)	−0.0007 (9)
C51	0.0340 (7)	0.0413 (8)	0.0452 (8)	−0.0052 (6)	0.0031 (6)	−0.0012 (6)
C52	0.0376 (7)	0.0427 (8)	0.0460 (9)	−0.0031 (6)	0.0022 (7)	−0.0022 (7)
C53	0.0457 (9)	0.0464 (9)	0.0641 (11)	−0.0030 (7)	0.0024 (8)	−0.0120 (8)
C54	0.0522 (10)	0.0400 (9)	0.0847 (15)	0.0039 (8)	0.0012 (10)	−0.0012 (10)
C55	0.0496 (10)	0.0566 (11)	0.0681 (13)	0.0019 (8)	−0.0053 (9)	0.0157 (10)
C56	0.0477 (9)	0.0507 (10)	0.0485 (9)	−0.0008 (8)	−0.0036 (7)	0.0002 (8)
C57	0.0377 (8)	0.0456 (9)	0.0406 (8)	0.0007 (6)	0.0024 (6)	−0.0021 (7)
C58	0.0450 (9)	0.0589 (10)	0.0455 (9)	−0.0052 (8)	0.0038 (7)	0.0034 (8)
C59	0.0495 (10)	0.0808 (14)	0.0509 (10)	0.0084 (9)	0.0144 (8)	0.0179 (10)
C60	0.0659 (13)	0.0934 (17)	0.0424 (10)	0.0231 (11)	0.0126 (9)	−0.0017 (10)
C61	0.0809 (15)	0.0694 (13)	0.0588 (12)	0.0157 (11)	0.0042 (11)	−0.0224 (10)
C62	0.0624 (11)	0.0503 (10)	0.0542 (10)	−0.0046 (8)	0.0098 (9)	−0.0105 (8)
C63	0.0451 (8)	0.0475 (9)	0.0350 (8)	0.0022 (7)	0.0078 (6)	−0.0016 (7)
C64	0.0425 (8)	0.0525 (10)	0.0444 (8)	−0.0008 (7)	0.0054 (7)	0.0036 (7)
C65	0.0460 (10)	0.0729 (13)	0.0487 (10)	−0.0068 (9)	0.0022 (8)	0.0031 (9)
C66	0.0563 (11)	0.0865 (15)	0.0534 (11)	0.0114 (11)	−0.0002 (9)	0.0127 (10)
C67	0.0757 (14)	0.0601 (12)	0.0577 (12)	0.0196 (10)	0.0037 (10)	0.0054 (10)
C68	0.0663 (11)	0.0509 (10)	0.0508 (10)	0.0108 (9)	0.0002 (9)	−0.0057 (8)

### Geometric parameters (Å, °)

**Table:**

P1—C9	1.7900 (19)	C23—H23A	0.9500
P1—C5	1.7905 (18)	C24—C25	1.379 (4)
P1—C1	1.791 (2)	C24—H24A	0.9500
P1—C2	1.8033 (17)	C25—C26	1.396 (3)
P2—C4	1.7846 (19)	C25—H25A	0.9500
P2—C13	1.7926 (18)	C26—H26A	0.9500
P2—C17	1.7959 (17)	C27—C28	1.397 (3)
P2—C3	1.8017 (16)	C27—C32	1.399 (3)
O1—C7	1.400 (3)	C28—C29	1.418 (4)
O1—C8	1.411 (3)	C28—H28A	0.9500
O2—C11	1.405 (3)	C29—C30	1.360 (5)
O2—C12	1.408 (3)	C29—H29A	0.9500
O3—C16	1.400 (3)	C30—C31	1.361 (5)
O3—C15	1.404 (3)	C30—H30A	0.9500
O4—C19	1.410 (2)	C31—C32	1.387 (4)
O4—C20	1.421 (3)	C31—H31A	0.9500
B1—C39	1.642 (3)	C32—H32A	0.9500
B1—C21	1.644 (2)	C33—C34	1.400 (2)
B1—C27	1.644 (3)	C33—C38	1.403 (2)
B1—C33	1.646 (2)	C34—C35	1.396 (3)
B2—C51	1.642 (2)	C34—H34A	0.9500
B2—C45	1.644 (2)	C35—C36	1.374 (3)
B2—C63	1.645 (2)	C35—H35A	0.9500
B2—C57	1.646 (2)	C36—C37	1.385 (3)
C1—H1A	0.9800	C36—H36A	0.9500
C1—H1B	0.9800	C37—C38	1.391 (2)
C1—H1C	0.9800	C37—H37A	0.9500
C2—C3	1.541 (2)	C38—H38A	0.9500
C2—H2A	0.9900	C39—C40	1.388 (3)
C2—H2B	0.9900	C39—C44	1.402 (3)
C3—H3A	0.9900	C40—C41	1.400 (3)
C3—H3B	0.9900	C40—H40A	0.9500
C4—H4A	0.9800	C41—C42	1.374 (4)
C4—H4B	0.9800	C41—H41A	0.9500
C4—H4C	0.9800	C42—C43	1.366 (4)
C5—C6	1.521 (3)	C42—H42A	0.9500
C5—H5A	0.9900	C43—C44	1.383 (3)
C5—H5B	0.9900	C43—H43A	0.9500
C6—C7	1.504 (3)	C44—H44A	0.9500
C6—H6A	0.9900	C45—C50	1.397 (3)
C6—H6B	0.9900	C45—C46	1.406 (3)
C7—H7A	0.9900	C46—C47	1.401 (3)
C7—H7B	0.9900	C46—H46A	0.9500
C8—H8A	0.9800	C47—C48	1.374 (4)
C8—H8B	0.9800	C47—H47A	0.9500
C8—H8C	0.9800	C48—C49	1.365 (4)
C9—C10	1.530 (3)	C48—H48A	0.9500
C9—H9A	0.9900	C49—C50	1.404 (3)
C9—H9B	0.9900	C49—H49A	0.9500
C10—C11	1.501 (3)	C50—H50A	0.9500
C10—H10A	0.9900	C51—C56	1.391 (2)
C10—H10B	0.9900	C51—C52	1.406 (2)
C11—H11A	0.9900	C52—C53	1.391 (2)
C11—H11B	0.9900	C52—H52A	0.9500
C12—H12A	0.9800	C53—C54	1.378 (3)
C12—H12B	0.9800	C53—H53A	0.9500
C12—H12C	0.9800	C54—C55	1.376 (3)
C13—C14	1.518 (2)	C54—H54A	0.9500
C13—H13A	0.9900	C55—C56	1.399 (3)
C13—H13B	0.9900	C55—H55A	0.9500
C14—C15	1.517 (3)	C56—H56A	0.9500
C14—H14A	0.9900	C57—C58	1.397 (2)
C14—H14B	0.9900	C57—C62	1.397 (2)
C15—H15A	0.9900	C58—C59	1.379 (3)
C15—H15B	0.9900	C58—H58A	0.9500
C16—H16A	0.9800	C59—C60	1.382 (3)
C16—H16B	0.9800	C59—H59A	0.9500
C16—H16C	0.9800	C60—C61	1.379 (3)
C17—C18	1.532 (2)	C60—H60A	0.9500
C17—H17A	0.9900	C61—C62	1.392 (3)
C17—H17B	0.9900	C61—H61A	0.9500
C18—C19	1.518 (3)	C62—H62A	0.9500
C18—H18A	0.9900	C63—C64	1.395 (2)
C18—H18B	0.9900	C63—C68	1.397 (3)
C19—H19A	0.9900	C64—C65	1.390 (3)
C19—H19B	0.9900	C64—H64A	0.9500
C20—H20A	0.9800	C65—C66	1.374 (3)
C20—H20B	0.9800	C65—H65A	0.9500
C20—H20C	0.9800	C66—C67	1.371 (3)
C21—C26	1.399 (2)	C66—H66A	0.9500
C21—C22	1.408 (3)	C67—C68	1.392 (3)
C22—C23	1.388 (3)	C67—H67A	0.9500
C22—H22A	0.9500	C68—H68A	0.9500
C23—C24	1.370 (4)		
			
C9—P1—C5	109.79 (9)	H20A—C20—H20C	109.5
C9—P1—C1	111.15 (10)	H20B—C20—H20C	109.5
C5—P1—C1	110.57 (10)	C26—C21—C22	115.68 (15)
C9—P1—C2	108.97 (8)	C26—C21—B1	122.17 (15)
C5—P1—C2	108.32 (9)	C22—C21—B1	121.82 (16)
C1—P1—C2	107.96 (9)	C23—C22—C21	122.1 (2)
C4—P2—C13	110.96 (9)	C23—C22—H22A	119.0
C4—P2—C17	112.44 (9)	C21—C22—H22A	119.0
C13—P2—C17	110.05 (9)	C24—C23—C22	120.7 (2)
C4—P2—C3	105.55 (9)	C24—C23—H23A	119.6
C13—P2—C3	107.64 (8)	C22—C23—H23A	119.6
C17—P2—C3	110.00 (8)	C23—C24—C25	119.07 (18)
C7—O1—C8	112.3 (2)	C23—C24—H24A	120.5
C11—O2—C12	114.17 (19)	C25—C24—H24A	120.5
C16—O3—C15	113.51 (16)	C24—C25—C26	120.5 (2)
C19—O4—C20	111.89 (17)	C24—C25—H25A	119.7
C39—B1—C21	103.73 (13)	C26—C25—H25A	119.7
C39—B1—C27	112.03 (14)	C25—C26—C21	121.92 (19)
C21—B1—C27	111.83 (14)	C25—C26—H26A	119.0
C39—B1—C33	114.07 (14)	C21—C26—H26A	119.0
C21—B1—C33	111.93 (13)	C28—C27—C32	115.8 (2)
C27—B1—C33	103.51 (13)	C28—C27—B1	120.61 (17)
C51—B2—C45	104.12 (13)	C32—C27—B1	123.42 (19)
C51—B2—C63	113.83 (14)	C27—C28—C29	121.3 (3)
C45—B2—C63	112.87 (14)	C27—C28—H28A	119.4
C51—B2—C57	111.73 (13)	C29—C28—H28A	119.4
C45—B2—C57	111.02 (13)	C30—C29—C28	120.8 (3)
C63—B2—C57	103.49 (12)	C30—C29—H29A	119.6
P1—C1—H1A	109.5	C28—C29—H29A	119.6
P1—C1—H1B	109.5	C29—C30—C31	118.5 (3)
H1A—C1—H1B	109.5	C29—C30—H30A	120.8
P1—C1—H1C	109.5	C31—C30—H30A	120.8
H1A—C1—H1C	109.5	C30—C31—C32	121.9 (3)
H1B—C1—H1C	109.5	C30—C31—H31A	119.1
C3—C2—P1	111.88 (11)	C32—C31—H31A	119.1
C3—C2—H2A	109.2	C31—C32—C27	121.7 (3)
P1—C2—H2A	109.2	C31—C32—H32A	119.2
C3—C2—H2B	109.2	C27—C32—H32A	119.2
P1—C2—H2B	109.2	C34—C33—C38	115.01 (15)
H2A—C2—H2B	107.9	C34—C33—B1	122.82 (14)
C2—C3—P2	114.83 (12)	C38—C33—B1	121.92 (14)
C2—C3—H3A	108.6	C35—C34—C33	122.48 (17)
P2—C3—H3A	108.6	C35—C34—H34A	118.8
C2—C3—H3B	108.6	C33—C34—H34A	118.8
P2—C3—H3B	108.6	C36—C35—C34	120.61 (18)
H3A—C3—H3B	107.5	C36—C35—H35A	119.7
P2—C4—H4A	109.5	C34—C35—H35A	119.7
P2—C4—H4B	109.5	C35—C36—C37	118.87 (17)
H4A—C4—H4B	109.5	C35—C36—H36A	120.6
P2—C4—H4C	109.5	C37—C36—H36A	120.6
H4A—C4—H4C	109.5	C36—C37—C38	120.05 (18)
H4B—C4—H4C	109.5	C36—C37—H37A	120.0
C6—C5—P1	114.76 (14)	C38—C37—H37A	120.0
C6—C5—H5A	108.6	C37—C38—C33	122.96 (17)
P1—C5—H5A	108.6	C37—C38—H38A	118.5
C6—C5—H5B	108.6	C33—C38—H38A	118.5
P1—C5—H5B	108.6	C40—C39—C44	115.23 (18)
H5A—C5—H5B	107.6	C40—C39—B1	123.43 (16)
C7—C6—C5	110.77 (18)	C44—C39—B1	121.20 (18)
C7—C6—H6A	109.5	C39—C40—C41	122.3 (2)
C5—C6—H6A	109.5	C39—C40—H40A	118.9
C7—C6—H6B	109.5	C41—C40—H40A	118.9
C5—C6—H6B	109.5	C42—C41—C40	119.9 (3)
H6A—C6—H6B	108.1	C42—C41—H41A	120.0
O1—C7—C6	108.13 (17)	C40—C41—H41A	120.0
O1—C7—H7A	110.1	C43—C42—C41	119.6 (2)
C6—C7—H7A	110.1	C43—C42—H42A	120.2
O1—C7—H7B	110.1	C41—C42—H42A	120.2
C6—C7—H7B	110.1	C42—C43—C44	119.9 (2)
H7A—C7—H7B	108.4	C42—C43—H43A	120.0
O1—C8—H8A	109.5	C44—C43—H43A	120.0
O1—C8—H8B	109.5	C43—C44—C39	123.0 (3)
H8A—C8—H8B	109.5	C43—C44—H44A	118.5
O1—C8—H8C	109.5	C39—C44—H44A	118.5
H8A—C8—H8C	109.5	C50—C45—C46	115.58 (16)
H8B—C8—H8C	109.5	C50—C45—B2	123.35 (17)
C10—C9—P1	115.04 (13)	C46—C45—B2	120.90 (15)
C10—C9—H9A	108.5	C47—C46—C45	123.04 (19)
P1—C9—H9A	108.5	C47—C46—H46A	118.5
C10—C9—H9B	108.5	C45—C46—H46A	118.5
P1—C9—H9B	108.5	C48—C47—C46	119.4 (2)
H9A—C9—H9B	107.5	C48—C47—H47A	120.3
C11—C10—C9	111.11 (17)	C46—C47—H47A	120.3
C11—C10—H10A	109.4	C49—C48—C47	119.3 (2)
C9—C10—H10A	109.4	C49—C48—H48A	120.4
C11—C10—H10B	109.4	C47—C48—H48A	120.4
C9—C10—H10B	109.4	C48—C49—C50	121.7 (2)
H10A—C10—H10B	108.0	C48—C49—H49A	119.2
O2—C11—C10	108.41 (16)	C50—C49—H49A	119.2
O2—C11—H11A	110.0	C45—C50—C49	121.0 (2)
C10—C11—H11A	110.0	C45—C50—H50A	119.5
O2—C11—H11B	110.0	C49—C50—H50A	119.5
C10—C11—H11B	110.0	C56—C51—C52	115.02 (15)
H11A—C11—H11B	108.4	C56—C51—B2	123.08 (15)
O2—C12—H12A	109.5	C52—C51—B2	121.57 (15)
O2—C12—H12B	109.5	C53—C52—C51	123.15 (17)
H12A—C12—H12B	109.5	C53—C52—H52A	118.4
O2—C12—H12C	109.5	C51—C52—H52A	118.4
H12A—C12—H12C	109.5	C54—C53—C52	119.55 (18)
H12B—C12—H12C	109.5	C54—C53—H53A	120.2
C14—C13—P2	116.06 (13)	C52—C53—H53A	120.2
C14—C13—H13A	108.3	C55—C54—C53	119.50 (17)
P2—C13—H13A	108.3	C55—C54—H54A	120.3
C14—C13—H13B	108.3	C53—C54—H54A	120.3
P2—C13—H13B	108.3	C54—C55—C56	120.12 (18)
H13A—C13—H13B	107.4	C54—C55—H55A	119.9
C15—C14—C13	109.46 (16)	C56—C55—H55A	119.9
C15—C14—H14A	109.8	C51—C56—C55	122.63 (18)
C13—C14—H14A	109.8	C51—C56—H56A	118.7
C15—C14—H14B	109.8	C55—C56—H56A	118.7
C13—C14—H14B	109.8	C58—C57—C62	115.15 (16)
H14A—C14—H14B	108.2	C58—C57—B2	121.35 (15)
O3—C15—C14	107.17 (15)	C62—C57—B2	122.96 (15)
O3—C15—H15A	110.3	C59—C58—C57	123.00 (19)
C14—C15—H15A	110.3	C59—C58—H58A	118.5
O3—C15—H15B	110.3	C57—C58—H58A	118.5
C14—C15—H15B	110.3	C58—C59—C60	120.36 (19)
H15A—C15—H15B	108.5	C58—C59—H59A	119.8
O3—C16—H16A	109.5	C60—C59—H59A	119.8
O3—C16—H16B	109.5	C61—C60—C59	118.69 (18)
H16A—C16—H16B	109.5	C61—C60—H60A	120.7
O3—C16—H16C	109.5	C59—C60—H60A	120.7
H16A—C16—H16C	109.5	C60—C61—C62	120.2 (2)
H16B—C16—H16C	109.5	C60—C61—H61A	119.9
C18—C17—P2	112.83 (12)	C62—C61—H61A	119.9
C18—C17—H17A	109.0	C61—C62—C57	122.56 (19)
P2—C17—H17A	109.0	C61—C62—H62A	118.7
C18—C17—H17B	109.0	C57—C62—H62A	118.7
P2—C17—H17B	109.0	C64—C63—C68	114.39 (16)
H17A—C17—H17B	107.8	C64—C63—B2	125.48 (15)
C19—C18—C17	111.70 (15)	C68—C63—B2	119.92 (16)
C19—C18—H18A	109.3	C65—C64—C63	123.26 (18)
C17—C18—H18A	109.3	C65—C64—H64A	118.4
C19—C18—H18B	109.3	C63—C64—H64A	118.4
C17—C18—H18B	109.3	C66—C65—C64	120.2 (2)
H18A—C18—H18B	107.9	C66—C65—H65A	119.9
O4—C19—C18	108.01 (14)	C64—C65—H65A	119.9
O4—C19—H19A	110.1	C67—C66—C65	118.7 (2)
C18—C19—H19A	110.1	C67—C66—H66A	120.7
O4—C19—H19B	110.1	C65—C66—H66A	120.7
C18—C19—H19B	110.1	C66—C67—C68	120.5 (2)
H19A—C19—H19B	108.4	C66—C67—H67A	119.8
O4—C20—H20A	109.5	C68—C67—H67A	119.8
O4—C20—H20B	109.5	C67—C68—C63	122.9 (2)
H20A—C20—H20B	109.5	C67—C68—H68A	118.5
O4—C20—H20C	109.5	C63—C68—H68A	118.5
			
C9—P1—C2—C3	63.90 (15)	B1—C33—C38—C37	−176.12 (15)
C5—P1—C2—C3	−55.50 (15)	C21—B1—C39—C40	99.06 (17)
C1—P1—C2—C3	−175.26 (14)	C27—B1—C39—C40	−140.16 (16)
P1—C2—C3—P2	−168.31 (9)	C33—B1—C39—C40	−23.0 (2)
C4—P2—C3—C2	−175.78 (14)	C21—B1—C39—C44	−76.36 (19)
C13—P2—C3—C2	−57.21 (15)	C27—B1—C39—C44	44.4 (2)
C17—P2—C3—C2	62.69 (15)	C33—B1—C39—C44	161.61 (15)
C9—P1—C5—C6	64.28 (16)	C44—C39—C40—C41	−1.0 (2)
C1—P1—C5—C6	−58.72 (17)	B1—C39—C40—C41	−176.65 (16)
C2—P1—C5—C6	−176.83 (13)	C39—C40—C41—C42	−1.1 (3)
P1—C5—C6—C7	167.84 (15)	C40—C41—C42—C43	2.0 (3)
C8—O1—C7—C6	177.5 (3)	C41—C42—C43—C44	−0.7 (3)
C5—C6—C7—O1	−63.3 (2)	C42—C43—C44—C39	−1.6 (3)
C5—P1—C9—C10	178.90 (13)	C40—C39—C44—C43	2.3 (3)
C1—P1—C9—C10	−58.45 (16)	B1—C39—C44—C43	178.10 (17)
C2—P1—C9—C10	60.41 (16)	C51—B2—C45—C50	−103.17 (19)
P1—C9—C10—C11	−166.78 (14)	C63—B2—C45—C50	20.8 (2)
C12—O2—C11—C10	178.28 (19)	C57—B2—C45—C50	136.45 (18)
C9—C10—C11—O2	66.7 (2)	C51—B2—C45—C46	71.88 (18)
C4—P2—C13—C14	−51.49 (17)	C63—B2—C45—C46	−164.18 (15)
C17—P2—C13—C14	73.60 (16)	C57—B2—C45—C46	−48.5 (2)
C3—P2—C13—C14	−166.52 (14)	C50—C45—C46—C47	−0.4 (3)
P2—C13—C14—C15	175.32 (14)	B2—C45—C46—C47	−175.86 (17)
C16—O3—C15—C14	172.34 (18)	C45—C46—C47—C48	0.3 (3)
C13—C14—C15—O3	−60.0 (2)	C46—C47—C48—C49	0.1 (4)
C4—P2—C17—C18	−60.58 (15)	C47—C48—C49—C50	−0.4 (4)
C13—P2—C17—C18	175.18 (12)	C46—C45—C50—C49	0.2 (3)
C3—P2—C17—C18	56.74 (15)	B2—C45—C50—C49	175.5 (2)
P2—C17—C18—C19	−166.12 (13)	C48—C49—C50—C45	0.2 (4)
C20—O4—C19—C18	175.65 (18)	C45—B2—C51—C56	−91.44 (18)
C17—C18—C19—O4	63.59 (19)	C63—B2—C51—C56	145.24 (15)
C39—B1—C21—C26	−83.54 (18)	C57—B2—C51—C56	28.5 (2)
C27—B1—C21—C26	155.54 (16)	C45—B2—C51—C52	81.63 (18)
C33—B1—C21—C26	39.9 (2)	C63—B2—C51—C52	−41.7 (2)
C39—B1—C21—C22	89.48 (19)	C57—B2—C51—C52	−158.47 (14)
C27—B1—C21—C22	−31.4 (2)	C56—C51—C52—C53	−1.6 (2)
C33—B1—C21—C22	−147.08 (16)	B2—C51—C52—C53	−175.24 (15)
C26—C21—C22—C23	−0.9 (3)	C51—C52—C53—C54	1.6 (3)
B1—C21—C22—C23	−174.32 (18)	C52—C53—C54—C55	−0.8 (3)
C21—C22—C23—C24	−0.9 (3)	C53—C54—C55—C56	0.2 (3)
C22—C23—C24—C25	1.9 (3)	C52—C51—C56—C55	1.0 (2)
C23—C24—C25—C26	−1.0 (3)	B2—C51—C56—C55	174.53 (16)
C24—C25—C26—C21	−0.9 (3)	C54—C55—C56—C51	−0.4 (3)
C22—C21—C26—C25	1.8 (2)	C51—B2—C57—C58	45.8 (2)
B1—C21—C26—C25	175.18 (16)	C45—B2—C57—C58	161.53 (16)
C39—B1—C27—C28	−165.90 (17)	C63—B2—C57—C58	−77.12 (19)
C21—B1—C27—C28	−49.9 (2)	C51—B2—C57—C62	−143.08 (17)
C33—B1—C27—C28	70.7 (2)	C45—B2—C57—C62	−27.3 (2)
C39—B1—C27—C32	19.0 (2)	C63—B2—C57—C62	94.03 (19)
C21—B1—C27—C32	134.94 (17)	C62—C57—C58—C59	−0.8 (3)
C33—B1—C27—C32	−104.38 (18)	B2—C57—C58—C59	171.04 (17)
C32—C27—C28—C29	0.8 (3)	C57—C58—C59—C60	0.7 (3)
B1—C27—C28—C29	−174.7 (2)	C58—C59—C60—C61	0.0 (3)
C27—C28—C29—C30	−2.2 (4)	C59—C60—C61—C62	−0.4 (3)
C28—C29—C30—C31	1.7 (4)	C60—C61—C62—C57	0.3 (3)
C29—C30—C31—C32	0.0 (4)	C58—C57—C62—C61	0.3 (3)
C30—C31—C32—C27	−1.4 (3)	B2—C57—C62—C61	−171.36 (18)
C28—C27—C32—C31	0.9 (3)	C51—B2—C63—C64	−15.1 (2)
B1—C27—C32—C31	176.26 (18)	C45—B2—C63—C64	−133.53 (16)
C39—B1—C33—C34	145.90 (15)	C57—B2—C63—C64	106.37 (17)
C21—B1—C33—C34	28.5 (2)	C51—B2—C63—C68	170.53 (15)
C27—B1—C33—C34	−92.10 (17)	C45—B2—C63—C68	52.1 (2)
C39—B1—C33—C38	−40.2 (2)	C57—B2—C63—C68	−67.99 (19)
C21—B1—C33—C38	−157.58 (15)	C68—C63—C64—C65	−2.1 (2)
C27—B1—C33—C38	81.82 (18)	B2—C63—C64—C65	−176.73 (16)
C38—C33—C34—C35	1.4 (2)	C63—C64—C65—C66	2.4 (3)
B1—C33—C34—C35	175.75 (16)	C64—C65—C66—C67	−0.7 (3)
C33—C34—C35—C36	−0.5 (3)	C65—C66—C67—C68	−1.0 (3)
C34—C35—C36—C37	−0.3 (3)	C66—C67—C68—C63	1.2 (3)
C35—C36—C37—C38	0.0 (3)	C64—C63—C68—C67	0.3 (3)
C36—C37—C38—C33	1.1 (3)	B2—C63—C68—C67	175.25 (17)
C34—C33—C38—C37	−1.8 (2)		
